# S67. INTEGRATION OF SENSORY AND SOCIAL INFORMATION DURING DECISION MAKING IN SCHIZOPHRENIA

**DOI:** 10.1093/schbul/sby018.854

**Published:** 2018-04-01

**Authors:** Arndis Simonsen, Riccardo Fusaroli, Vibeke Bliksted, Ole Mors, Andreas Roepstorff, Daniel Campbell-Meiklejohn

**Affiliations:** 1 The Lundbeck Foundation Initiative for Integrative Psychiatric Research, Aarhus University; 2 Aarhus University; 3 Aarhus University Hospital; 4 The Lundbeck Foundation Initiative for Integrative Psychiatric Research, Aarhus University Hospital; 5 University of Sussex

## Abstract

**Background:**

Previous findings suggest that schizophrenia is associated with abnormalities in information integration. The recent Bayesian model of circular inference attempts to characterize the information integration style in schizophrenia (Jardri et al. (2017). Nat Commun, 8:14218). The model suggests that during information integration of prior beliefs and sensory evidence, patients tend to put too much weight on the sensory evidence and to take it into account multiple times (over-count) compared to healthy individuals. An imbalance in excitatory/inhibitory regulation of hierarchical neural processing has been suggested to cause of this phenomenon (Jardri et al., 2017). Here, we investigated whether circular inference could be extended to describe the integration of sensory information and socially acquired information and whether the specific information integration style could contribute to the characteristic symptomatology and the social impairments seen in the disorder.

**Methods:**

Thirty-five patients with schizophrenia or schizo-affective disorder and 40 matched healthy controls performed the task. Participants had to guess the color (red or green) of the next marble to be drawn from a hidden urn based on information from their own sample (eight marbles) and the choices and confidence (high or low) of four other people. We fitted and comparatively assessed four multilevel Bayesian models (generalized linear model, simple Bayes, weighted Bayes, circular inference) describing how patients and controls integrated information. Positive and negative symptom severity and social functioning were also assessed.

**Results:**

The circular inference model best described the information integration in both patients and controls (WAIC weight = 1). Patients tended to over-weigh (β = 0.48, 95% CI: 0.30; 0.62) and over-count (β = 0.30, 95% CI: 0.12; 0.47) sensory information and under-weigh (β = -0.29, 95% CI: -0.42; -0.13) and under-count (β = -0.23, 95% CI: -0.35; -0.06) social information compared to controls. Crucially, this varied with symptomatology: the higher the symptom severity, the more over-counting and the higher the weight on sensory information and the more under-counting and the less weight on social information. More weight on social information was associated with higher level of functioning in the patients (β = 0.88, 95% CI: 0.01; 3.73).

**Discussion:**

All participants integrated social and sensory information in a non-linear fashion. Patients displayed a distinctive tendency to rely more and less discriminatively on sensory than on social information. This information integration style may contribute to the characteristic symptoms and the social impairments in schizophrenia. Further exploration of this potential causal role is warranted.

